# Mathematical Modeling in Neuroscience: Neuronal Activity and Its Modulation by Astrocytes

**DOI:** 10.3389/fnint.2016.00003

**Published:** 2016-02-04

**Authors:** Shivendra G. Tewari, Manoj K. Gottipati, Vladimir Parpura

**Affiliations:** ^1^Molecular and Integrative Physiology, University of MichiganAnn Arbor, MI, USA; ^2^Department of Neurobiology, University of Alabama at BirminghamBirmingham, AL, USA

**Keywords:** neurons, astrocytes, mathematical model, parameter estimation

Research in neuroscience has come a long way since it was first hypothesized, in the early twentieth century, that dynamic changes in ion permeability underlie an event termed as action potential (Bernstein, 1912). Research along the same lines in the 1950s by Hodgkin and Huxley (1952) elucidated the dependence of action potential on the permeability of potassium and sodium ions—a theory achieved using quantitative analysis of potassium, sodium, and leak currents. Using mathematical modeling, they suggested that potassium and sodium conduits exist in distinct states (open, closed, or inactive) during an action potential; this was at a time when the composition of excitable membrane was largely unknown. Their mathematical model revolutionized the field of neurobiology and still forms the basis for many of the current mathematical models. Over the past several years, as more information on different channel types became available, more complex neuronal action potential models accounting for several channel types have been built (Traub et al., 1994; Bower and Beeman, 1995); a comparison between the original Hodgkin-Huxley model and a more detailed contemporary model is shown in Figures 1A–C.

**Figure 1:**
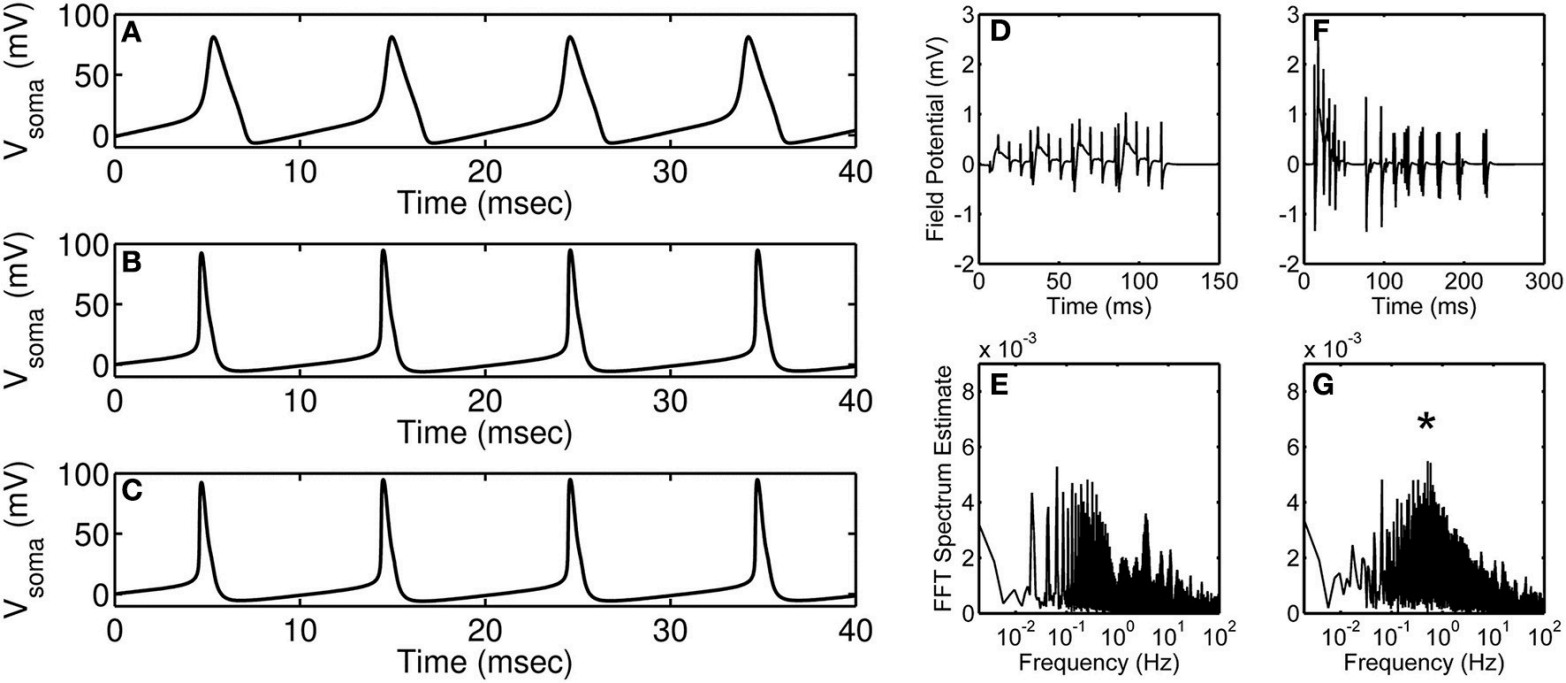
**Modeling of action potential discharges and the effect of astrocyte on accumulated neural discharges**. **(A)** Hodgkin-Huxley squid axon model (Hodgkin and Huxley, 1952) injected with 35 μA·cm^−2^ current. **(B,C)** Branching dendrite model of a rodent CA3 pyramidal neuron (Traub et al., 1994) injected with 15 nA current into the soma compartment; **(B)** Control (wild-type) neuron modeling, and **(C)** Modeling of action potential discharges of a neuron with calcium-activated potassium channel knocked out. Simulations were obtained using published models and parameters. Model simulated field potentials of CA1 pyramidal neurons in the absence **(D)** and the presence **(F)** of peri-synaptic astrocyte. FFT spectrum analysis of the model simulated field potentials in the absence **(E)** and the presence **(G)** of astrocyte. **(D–G)** are modified and reproduced from Tewari and Parpura (2013). Asterisk denotes the astrocyte induced peak in the FFT spectrum.

With the hindsight, detailed models have been successful in emulating neuronal firing patterns observed *in vivo* or *in situ*. Primarily, computation of such detailed models has been possible due to significant technological advances that help solve differential equations in multiple dimensions. On one hand, detailed mathematical models are necessary to account for all the known proteins but on the other hand these detailed models possess redundant parameters which will lead to an unnecessary increase in the cost of computation. For example, consider the situation in Figures 1B,C where a detailed hippocampal CA3 region pyramidal neuron model is simulated under control conditions (B) and by knocking out its calcium-activated potassium channel (C). It is apparent from the figure that under both the conditions the action potential generated is unchanged, which indicates that incorporating all of the “known” proteins need not necessarily lead to a significant change in the model output of interest (in this case, an action potential). It is worth mentioning that the model described above involves only 897 ordinary differential equations, which is far less than the number of equations in a model accommodating all the neurons in the CA3-CA1 region [estimated to be ~20 × 10^6^ (West and Gundersen, 1990)]; the amount of redundancy in the latter model could be overwhelming and pruning the less significant proteins would aide in generating a region specific or whole-brain simulation. Of course, it is a “brigade” of interconnected neurons (identified experimentally by local field potential recordings), but not a single neuron *per se*, that plays a role in performing a task (Miller and Cohen, 2001; Buschman et al., 2012). This may contribute to an additional level of redundancy that needs to be assessed and optimized in order to successfully model the CA3-CA1 region.

An important player left out of the discussion above is the peri-synaptic astrocyte. In recent years, it has been demonstrated that the astrocytes can: (1) facilitate or depress synaptic plasticity (De Pittà et al., 2015), (2) synchronize CA1 neuronal firing (Fellin et al., 2004), (3) modulate extracellular field potentials (Lee et al., 2014), (4) repair damaged synapses (Wade et al., 2012), and/or (5) initiate epileptic discharges (Reato et al., 2012; Tewari and Parpura, 2013). It has also been hypothesized that astrocytes can (1) store memories (Caudle, 2006), (2) promote motor-skill learning (Padmashri et al., 2015), and (3) modulate sleep (Halassa et al., 2009). Considering this overwhelming evidence of the involvement of astrocytes in brain activity, it has become important to integrate the effect of astrocyte signaling while simulating region specific neural oscillations or whole-brain rhythms. In the past decade, efforts have been made to integrate detailed models of different neurons on a large scale to mimic brain region specific neural oscillation patterns (Traub et al., 2005; Reimann et al., 2013), however, astrocytes have been left out of their calculations.

Recently, there have been attempts to include astrocytes in modeling of synaptic transmission. For example, Figures 1D–G show simulations from Tewari and Parpura (2013) performed in the absence (Figures 1D,E) and the presence (Figures 1F,G) of astrocyte signaling. Briefly, these model simulations show an effect of extra-synaptic signaling on the amplitude and the frequency of neural oscillations. The amplitude of neural oscillations is represented by field potentials and the frequency of neural oscillations is estimated using fast Fourier transform (FFT). It is apparent from these simulations that the presence of astrocyte increases the amplitude of neural oscillations (compare Figure 1D and Figure 1F) and also orchestrates neural firing to occur at a certain frequency (note “asterisk” in Figure 1G). Note that these simulations were performed under minimal recurrent synapses within a neural population which suggests that both neural oscillation amplitude and frequency would change depending upon astrocyte input.

Two types of modeling issues have been introduced above, (1.) which arises due to redundant model parameters and (2.) which arises due to the structural limitations of the model (e.g., ignoring glio-transmission in a model of CA3-CA1 region). To avoid these issues, as mentioned earlier, two things need to be considered before even initiating mathematical analysis of an experimental dataset:

- The extent to which a given mathematical model can mimic a biological response.
- Level of details that can be ignored in an experimental data-set without significant loss in the biological question being addressed.

Action potentials generated using the famous Hodgkin-Huxley model (shown in Figure 1A) could be considered today as the case (2) as some of the details we know today are not included in the model. However, it is noteworthy that the time taken to simulate the Hodgkin-Huxley model is more than 500 times less as compared to the more elaborate Traub model (shown in Figure 1B), which can be considered as the case (1). Although the action potentials generated using these two models are noticeably different, there are methods available that can help build on (improve) the Hodgkin-Huxley model in a parsimonious way or reduce the Traub model to be computationally less expensive. The first step toward building a new model (or modifying an old model) should be to gather as much data as possible (from literature and/or experiments). The models developed should be checked for parsimony using Akaike information criterion (Bozdogan, 1987) or Bayesian information criterion (Neath and Cavanaugh, 2012). The best practice for estimating the parameters is to use a global optimization (Vinnakota and Bugenhagen, 2013) technique along with a local optimization technique (Byrd et al., 1999). Moreover, sensitivity analysis methods are very useful in identifying sensitive or insensitive parameters to facilitate model reduction (Saltelli et al., 2008), and should be applied before parameter estimation.

Future models of synaptic plasticity need to integrate the effect of astrocytes on the frequency of neural synchrony in different brain regions. It is understandable that prediction of region specific firing patterns would involve the integration of all possible neuron-astrocyte-neuron interactions in the region of interest which can be computationally challenging. For example, simulation of the network model presented in Figures 1D–G takes about 48 h on a laptop computer. Therefore, it is really important to employ modeling strategies which lead to simplified, computationally tractable and biologically relevant mathematical models.

## Author contributions

All authors listed, have made substantial, direct and intellectual contribution to the work, and approved it for publication.

### Conflict of interest statement

The authors declare that the research was conducted in the absence of any commercial or financial relationships that could be construed as a potential conflict of interest.
